# Bioactive Surfaces vs. Conventional Surfaces in Titanium Dental Implants: A Comparative Systematic Review

**DOI:** 10.3390/jcm9072047

**Published:** 2020-06-29

**Authors:** Nansi López-Valverde, Javier Flores-Fraile, Juan Manuel Ramírez, Bruno Macedo de Sousa, Silvia Herrero-Hernández, Antonio López-Valverde

**Affiliations:** 1 Department of Surgery, University of Salamanca, Instituto de Investigación Biomédica de Salamanca (IBSAL), 37007 Salamanca, Spain; nlovalher@usal.es (N.L.-V.); j.flores@usal.es (J.F.-F.); silvia_herrero@hotmail.com (S.H.-H.); 2 Department of Morphological Sciences, University of Cordoba, Avenida Menéndez Pidal s/n, 14071 Cordoba, Spain; jmramirez@uco.es; 3 Institute for Occlusion and Orofacial Pain Faculty of Medicine, University of Coimbra, Polo I - Edifício Central Rua Larga, 3004-504 Coimbra, Portugal; brunomsousa@usal.es

**Keywords:** Ti dental implants, bioactive surfaces, biofunctionalized surfaces, osseointegration

## Abstract

Animal studies and the scarce clinical trials available that have been conducted suggest that bioactive surfaces on dental implants could improve the osseointegration of such implants. The purpose of this systematic review was to compare the effectiveness of osseointegration of titanium (Ti) dental implants using bioactive surfaces with that of Ti implants using conventional surfaces such as sandblasted large-grit acid-etched (SLA) or similar surfaces. Applying the guidelines of the Preferred Reporting Items for Systematic Reviews and Meta-analyses (PRISMA) statement, the MEDLINE, PubMed Central and Web of Science databases were searched for scientific articles in April 2020. The keywords used were “dental implants”, “bioactive surfaces”, “biofunctionalized surfaces”, and “osseointegration”, according to the question: “Do bioactive dental implant surfaces have greater osseointegration capacity compared with conventional implant surfaces?” Risk of bias was assessed using the Cochrane Collaboration tool. 128 studies were identified, of which only 30 met the inclusion criteria: 3 clinical trials and 27 animal studies. The average STROBE (STrengthening the Reporting of OBservational studies in Epidemiology) and ARRIVE (Animal Research: Reporting of In Vivo Experiments) scores were 15.13 ± 2.08 and 17.7±1.4, respectively. Implant stability quotient (ISQ) was reported in 3 studies; removal torque test (RTT)—in 1 study; intraoral periapical X-ray and microcomputed tomography radiological evaluation (RE)—in 4 studies; shear force (SF)—in 1 study; bone-to-implant contact (BIC)—in 12 studies; and BIC and bone area (BA) jointly—in 5 studies. All animal studies reported better bone-to-implant contact surface for bioactive surfaces as compared to control implants with a statistical significance of *p* < 0.05. Regarding the bioactive surfaces investigated, the best results were yielded by the one where mechanical and chemical treatment methods of the Ti surfaces were combined. Hydroxyapatite (HA) and calcium–phosphate (Ca–Ph) were the most frequently used bioactive surfaces. According to the results of this systematic review, certain bioactive surfaces have a positive effect on osseointegration, although certain coating biomolecules seem to influence early peri-implant bone formation. Further and more in-depth research in this field is required to reduce the time needed for osseointegration of dental implants.

## 1. Introduction

The concept of osseointegration was introduced by Brånemark et al. in 1969 [1] to be later defined by Albrektsson et al. as “a direct structural and functional connection between living bone and the surface of a load-bearing titanium (Ti) implant” [2]. Machined dental implant surfaces were the starting point [3] and, since then, different modifications to Ti surfaces have been tested in an attempt to improve the biological conditions and properties of osseointegration. Ti is a low bioactivity biomaterial, which is why different surface treatments have been developed aimed at improving osseointegration capacity [4].

Implant surface modification is one of the most novel and productive research fields acquiring relevance in the search for a system that meets the ideal functional and biological goals [5,6]. Surface topography plays a crucial role in osseointegration and it is known that cell response can be modulated by adapting implant surface texture. Rough micro-, submicro- and nanoscale topographies can be very effective in promoting osseointegration [7,8,9]. Some authors have reported that sandblasted and acid-etched (SLA) implants achieve osseointegration even in the absence of primary stability [8].

The main systems that have been developed and are used to achieve adequate implant surface roughness are sandblasting, acid etching, anodizing, and titanium plasma spraying [10]. Other strategies that have been proposed for the improvement of titanium surface osseointegration include coatings with hydroxyapatite, bioactive glasses, bisphosphonates, or collagen [11,12,13,14].

The bio-functionalization of a certain material consists of a modification of the physicochemical properties of its surface, which would allow an improvement in the biological response of an organism when it comes into contact with it.

Currently, most research is focused on antibacterial and antiadhesive surfaces which include both materials that are able to reduce bacterial adhesion on implant surfaces and active antibacterial materials with a defined antimicrobial activity [15,16].

**Figure 1:**
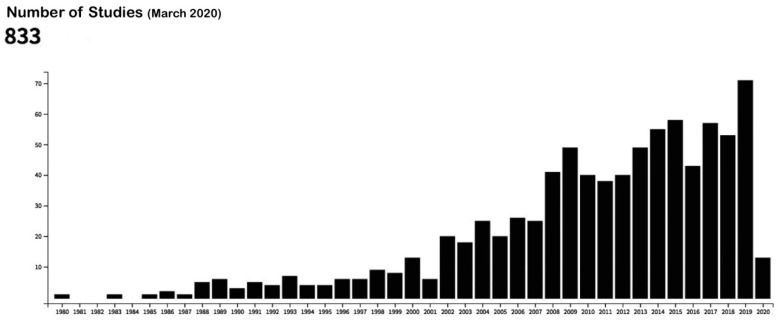
Publications in the US National Library of Medicine database with the following keywords: “bioactive surfaces” and “dental implants.” Source: US National Library of Medicine [18].

Bioactive surfaces are those capable of achieving a faster and better quality of osseointegration with the aim of solving such problems as poor bone quality or reducing waiting times for prosthetic loading [17]. Figure 1 provides a graph demonstrating the increase in publications regarding this topic in the last twenty years (elaborated with the data from the US National Library of Medicine).

Systematic reviews are an essential tool to synthesize the scientific information available, enhancing the validity of the findings of individual studies while at the same time detecting areas of uncertainty that require further research. Keeping this in mind, the purpose of this work was to conduct a systematic review of the literature comparing the effectiveness of osseointegration of bioactive dental implant surfaces with that of implants without such surface morphology.

## 2. Methods

We performed study selection according to the PRISMA (Preferred Reporting Items for Systematic Review and Meta-Analyses) guidelines for reporting systematic reviews and meta-analyses [19].

### 2.1. Protocol

The search strategy was conducted using the population, intervention, comparison, and outcome (PICO) framework based on the following question: “Do bioactive dental implant surfaces have greater osseointegration capacity compared with conventional implant surfaces?”

To answer this question, a sample population group of patients undergoing treatment with Ti dental implants with bioactive surfaces was selected. Controls were patients who were treated with conventional implant surfaces. The outcomes reviewed in the literature were the BIC (bone-to-implant contact), BA (bone area), RTT (removal torque test), RE (radiological evaluation), SF (shear force), or ISQ (implant stability quotient) values reported in the different selected studies.

### 2.2. Search Method for the Identification of Studies

A search in the MEDLINE, PubMed Central, and Web of Science electronic databases was conducted in April 2020 to identify relevant scientific articles. The search terms used were “Ti dental implants”, “bioactive surfaces”, “biofunctionalized surfaces”, and “osseointegration.”

### 2.3. Inclusion and Exclusion Criteria

Inclusion criteria:

(a) Studies published in English.
(b) Studies with Ti implants.
(c) Human studies with bioactive titanium implant placement procedures.
(d) Animal studies with bioactive titanium implant placement procedures.
(e) Studies evaluating BIC (bone-to-implant contact), BA (bone area), ISQ (implant stability quotient), RTT (removal torque test), RE (intraoral periapical X-ray and microcomputed tomography radiological evaluation), or SF (shear force).

Exclusion criteria:

(a) Studies using conventional Ti implants (SLA or similar surfaces).
(b) In vitro studies.
(c) Narrative reviews and systematic reviews.
(d) Case studies.
(e) Irrelevant (didactics, Delphi surveys…) and duplicate studies and those that did not meet the established inclusion criteria.

### 2.4. Data Extraction and Analysis

Studies that made no reference to the research question were removed and the titles and abstracts of the articles selected were obtained and entered in an Excel spreadsheet. Two reviewers (N.L.-V. and A.L.-V.) selected the titles and abstracts independently. Discrepancies in terms of study inclusion were discussed between the two mentioned reviewers until consensus was reached. Subsequently, full texts of the selected studies were obtained for their review and inclusion.

### 2.5. Risk of Bias (RoB) of Included Articles

The Cochrane Collaboration, London, UK, tool was used to assess methodology of the scientific evidence in all the selected studies as previously described [20].

### 2.6. Quality of the Reports of the Included Studies

This was assessed according to the modified STROBE statement (STrengthening the Reporting of OBservational studies in Epidemiology) (Table 1) [21] and ARRIVE guidelines (Animal Research: Reporting of In Vivo Experiments) (Table 2) [22], which include a total of 22 items. Each item was assessed by reviewers N.L.-V. and A.L.-V. who attributed scores of 0 (not reported) or 1 (reported) carrying out a complete count of all the studies included.

## 3. Results

### 3.1. Characteristics of the Studies

**Figure 2:**
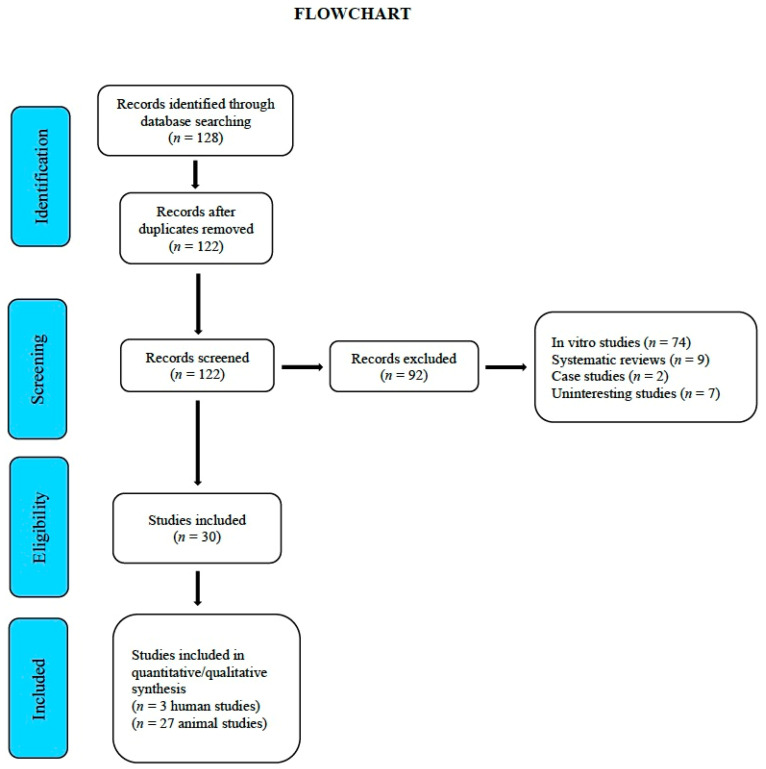
Flowchart.

By April 2020, a total of 128 studies had been gathered and subsequently assessed by the reviewers. From these, 92 studies were removed due to their being in vitro trials, duplicates, systematic reviews, or irrelevant, leaving a total of 30 studies: 27 were carried out on animals [26,27,28,29,30,31,32,33,34,35,36,37,38,39,40,41,42,43,44,45,46,47,48,49,50,51,52] and 3 on humans [23,24,25] (Figure 2 “Flowchart”). Table 3 and Table 4 provide a general description of the details of each study. Table S1 (PRISMA Checklist), describes section/items and pages.

### 3.2. ISQ, BIC, BA, RTT, RE, and SF Information

Of the clinical studies, only Gursoytrak and Ataoglu reported ISQ, finding no significant difference between Ti surfaces and bioactive (alkali-modified) surfaces after twelve weeks [23]; Malchiodi and colleagues [24] reported the BIC value by means of radiological analysis of the bone-implant interface in three biopsies in CaP-coated Ti implants; Mistry and colleagues [25] compared two modified Ti surfaces, one with HA and the other with bioactive glass, reporting bone-implant contact values obtained from CT scans (no significant differences).

In animal studies, ISQ was reported by 2 studies [26,27], both of which showed an increase when HA and alumina bioactive surfaces were used. BIC was reported in 11 studies [28,29,30,32,33,35,36,37,38,39,40,42,43,44,45,46,49,50,51]; BIC and BA together—5 studies [28,32,35,36,39]; RTT was reported in a study using rat tibiae where machined Ti surfaces and HA coated surfaces were compared [48]; RE (microcomputed tomography) is reported in 2 studies [31,34], SF—in one study [47].

### 3.3. Synthesis of Included Studies

Thirteen studies used rabbits as animal models [23,27,30,31,32,37,38,39,43,45,48,49], 5—pigs [28,34,41,44,51], 4—dogs [29,46,47,52], 3—rats [33,35,42], 2—goat and non-human primates [36,50]. Sixteen modifications of the Ti surface were used, the most used bioactive surface being HA [27,29,36,44,46,49]. In humans, the worst results were reported by Malchiodi and colleagues who studied a bioactive surface of CaP. The study of Germanier and colleagues [51] who compared SLA surfaces (sandblasted large-grit acid-etched) with surfaces modified by a bioactive peptide found significant differences in the extent of BIC during the early stages of bone regeneration (RGD (Arg–Gly–Asp)-SLA 61.68 ± 4.21, SLA 43.62 ± 10.79), reporting the highest statistical significance (*p* < 0.001) in terms of peri-implant bone growth among all the included studies. All animal studies [26,27,28,29,30,31,32,33,34,35,36,37,38,39,40,41,42,43,44,45,46,47,48,49,50,51,52] reported an increase in peri-implant bone formation when using the surfaces studied, as compared to control surfaces (*p* > 0.05), 6 of them reporting the best results (*p* < 0.01) in the formation of peri-implant bone in the surfaces studied [30,31,33,34,36,45].

### 3.4. Risk of Bias (RoB Cochrane Collaboration’s Tool)

**Figure 3:**
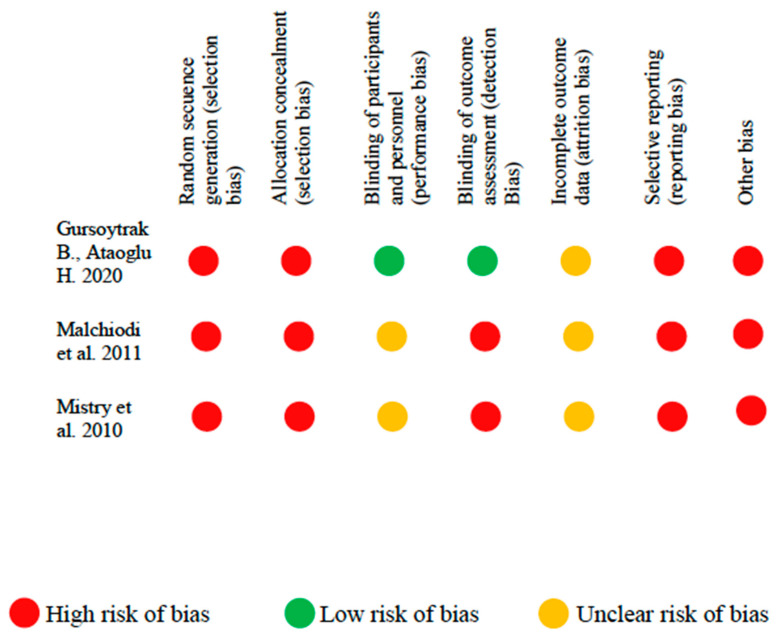
Risk of bias in clinical studies.

**Figure 4:**
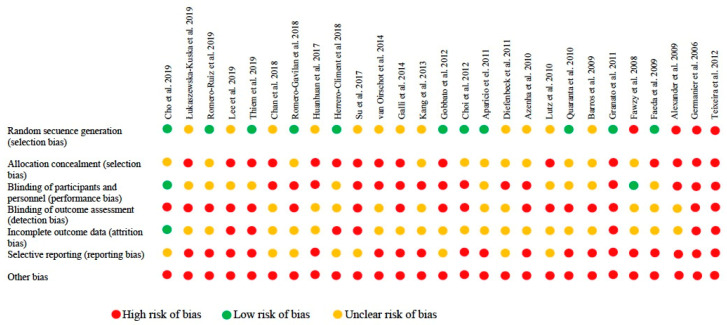
Risk of bias in animal studies.

In the studies considered is shown in Figure 3 and Figure 4. Among the clinical trials, only one [23] met the blinding of participants and personnel and the blinding of outcome assessment criteria. The random sequence generation and allocation concealment domains were not met in any of the clinical trials considered.

Among the animal studies, 46% met the random sequence generation domain and only 2 [26,48] met the blinding of participants and personnel criteria.

The average STROBE and ARRIVE scores were 15.13 ± 2.08 and 17.7 ± 1.4, respectively. According to the ARRIVE checklist, the study by Mistry et al. obtained the highest scores [25]; item 11 (accommodation and handling of animals) was only reported in 5 studies [26,27,28,29,30]; items 19, 20, and 21 (3Rs reported, adverse events, and study limitations) were not reported in any of the included studies. The maximum scores were achieved in the studies by Cho et al., 2019, Łukaszewska-Kuska et al., 2019, Romero-Ruiz et al., 2019, and Thiem et al., 2019 [26,27,28,29]. As regards the STROBE statement checklist, items 9 (bias), 14 (descriptive data), 19 (limitations), 20 (interpretation), and 21 (generalizability) were not reported in any of the studies (Table 3 and Table 4).

## 4. Discussion

The main purpose of implant surface modifications is to modulate the host tissue and favor osseointegration. This review assessed the capacity of improvement of osseointegration of bioactive surface modifications as compared to the conventional titanium surface (SLA or similar surfaces). Although a large part of research in implant dentistry is currently focused on the study of bioactive surfaces, only three studies based on human trials were found: one compared the ISQ of two types of implants, with and without a bioactive surface [23]; another studied a series of calcium phosphate (CaP)-coated Ti implants [24]; and the third study analyzed the effectiveness of hydroxyapatite and bioactive glass-coated Ti implants [25]. One of the reasons for the scarcity of this type of studies could be their ethical implications, since, for obvious ethical reasons, it is not possible to obtain gold standard histological samples (biopsies) to analyze bone formation around implants; however, one of the included clinical trials [24] studied through biopsy a series of samples obtained from patients. Nevertheless, even though bone-to-implant contact can be assessed using techniques such as electron microscopy [53,54], researchers have established histomorphometric analysis as the most widely used method in most studies [55,56].

The rest of the selected studies [26,27,28,29,30,31,32,33,34,35,36,37,38,39,40,41,42,43,44,45,46,47,48,49,50,51,52] are based on animal research using different animal models and a variety of Ti surface coatings, with HA and Ca–Ph being the most common [27,28,29,36,46,52]. These six studies provided remarkable results as regards osseointegration, with conclusive findings such as activation of osteoblastic activity and healing time through the increase of bone-to-implant interaction during the first 2 months after placement [29,49].

Hydroxyapatite (HA) is a non-inflammatory, non-toxic, and non-immunogenic material with osteoconductive and bioactive properties [57]. HA coating has been proposed for implant surface modification to promote bone healing and osseointegration, which would allow early functional loading. Nevertheless, there are currently no standard manufacturing guidelines for HA deposition on implant surfaces [58]. In the past, multiple failures with HA-coated implants were reported [59]; however, it seems that such failures could be due to poor quality coatings and product crystallization [60].

A systematic review conducted by Qadir and colleagues [57] affirms that the topography and chemical properties of amorphous HA coating surfaces influence cell behavior and ion-substituted HA coatings significantly increase cell adhesion, but can have a cytotoxic effect that slows the growth of the cells that are attached to the coating’s surface areas, however, some authors question the effectiveness of hydrophilic surfaces and HA-coated surfaces in terms of osteoblastic activation [29].

The healing times reported in the different studies included varied widely from 1 to 13 weeks [31,39]; in a study using goats, van Oirschot and colleagues [36] found that at 4 weeks, HA-coated Ti had an osseointegrating effect (BIC and BA values) superior to shot blasting/acid etching (grit-blasted/acid-etched implants); Faeda and colleagues [49] studied Ti surfaces modified through laser ablation and subsequently coated with HA measuring implant extraction force using RTT. The average removal torque was higher in HA-coated implants obtaining significant values (*p* = 0.05) at 4, 8, and 12 weeks and comparing them with control implants (only laser ablation implants and machined surface implants). It is also worth noting that Mistry and colleagues [25] found similar results between HA-coated implants and implants covered with bioactive glass in their clinical trial.

Another modification of the Ti surface coating used in the different studies included in this systematic review was calcium–phosphate (Ca–Ph) [23,39,42,45]. Bioactive Ca and Ph-based ceramics have received considerable attention over the years, leading to highly osteoconductive coatings [45,61]. In a study in rat tibiae, Diefenbeck and colleagues [42], using Ca–Ph-coated Ti implants, found a high rate of early osseointegration compared to conventional surface Ti implants. It should be noted that the best results in terms of BIC at 3, 4, and 8 weeks, were obtained with surfaces modified with Ca–Ph [45] despite the fact that certain authors exclusively attribute osteoconductive properties to it [62].

The highest statistical significance was found in the study by Germanier and colleagues [51] who compared conventional surfaces of Ti (SLA) and surfaces modified by a double peptide, RDG (Arg–Asp–Gly) and RGD (Arg–Gly–Asp). At 2 weeks, RGD-coated implants yielded significantly higher percentages of bone-to-implant contact than controls (*p* < 0.001). This RGD peptide could have osteogenic properties that correlated with effects that would alter cell binding and dissemination, generating a more differentiated cell morphology [63].

A large part of the included studies compared the surfaces studied with SLA or similar surfaces [26,27,28,29,30,33,34,41,46,51]. Bioactive and biofunctional concepts were unclear in the included studies, and only 9 of them [26,31,33,38,40,43,44,47,51] clearly specified biofunctionalization of the Ti surface.

New methods of surface preparation are currently under constant investigation. The successful osseointegration of dental implants depends on the amount of bone that is in direct contact with the implant surface. Destruction of the bone-implant-contact area (BIC) could lead to implant failure. Early osseointegration is influenced by the roughness and coatings of the Ti surfaces [64,65], however, infection is frequently the cause of failure of dental implants [66]. Implant infections are generally associated with Gram-negative periodontal pathogens (*Porphyromonas gingivalis*, *Prevotella intermedia*/*Prevotella nigrescens*, and *Actinobacillus actinomycetemcomitans*); various antimicrobial and antibiotic peptides are proposed in order to solve these drawbacks [67]. In contact with air, Ti undergoes an oxidation process that is of major importance in the osseointegration process; however, the oxide on the Ti surface includes a large number of impurities, which would hinder the osseointegration process [68].

Plasma biology is a new interdisciplinary research area that is currently being used to functionalize surfaces and improve their biocompatibility [69]. The relationship between plasma treatment of Ti surfaces and differentiation of bone tissue has been reported in several studies [70].

The objective of current technologies [71] is to generate thin plasmas using small and easy-to-use devices. Ujino and colleagues [72] showed that osteogenic adhesion and differentiation increased in the cells grown on plasma-treated Ti discs as compared to those raised on untreated discs. Their device uses piezoelectric mechanical resonance to amplify electrical energy and generate high voltage. In this way, it ionizes the surrounding atmospheric air and produces plasma. Conventional plasma devices require vacuum and processing is limited and expensive. In contrast, the Piezobrush^®^ PZ2 device (Relyon Plazma GmbH, Regensburg, Germany) used by Ujino and colleagues is compact and suitable for use in dental practices.

Because of its decontaminating properties, argon plasma (Ar), which is widely used as a coagulant in digestive surgery [73], has been proposed by certain authors [52,74,75,76,77] to improve early integration of Ti implants due to its decontaminating properties.

At low temperature, the Ar-oxygen plasma could be highly effective in cleaning surfaces, eliminating chemical residues, contaminants, and impurities in Ti, and producing an activating effect on the surface of the implant, which would improve cell proliferation and adhesion and, as a consequence, mineralization [74]; however, the devices used are expensive and work at high temperatures or low pressures, making them difficult to use in a regular dental office. Teixeira and colleagues [52] proposed its use in a dental office immediately before implantation through the use of non-thermal plasmas applied by means of manageable devices (KinPenTM^®^ device, INP, Greifswald, Germany) which allow the modification of the Ti surface at room temperature. Similarly, in a study in Beagle dogs, Giro and colleagues [78] reported significant effects in implants treated with low-temperature Ar plasma. Therefore, the low-temperature Ar plasma could be used directly in a dental office both for surface disinfection and for direct application, in root canal disinfection, or in other surgical techniques that are conducted immediately prior to implantation [79].

On the other hand, the experimental rodent models (rats and rabbits) used in many of the studies included, as well as the choice of implant location (tibia and femur), are not considered adequate models for the extrapolation of results to humans, among other reasons, because they lack cortical bone remodeling and because they stop growing much later than other mammals [80]. Additionally, the bones of rabbits, which are the most commonly used species in the studies included in this systematic review, are the most dissimilar in structure to human bone [81]. While none of the species meets all the requirements of an ideal model, understanding the differences in bone architecture and remodeling among the different experimental animal species could help researchers to select an adequate species for a specific research question.

Nonetheless, this systematic review is not free from limitations as regards number, quality, and methodology of the studies included, both animal and human. First, only three clinical trials were found [23,24,25], which is insufficient to confirm the results they describe as significant in humans. Second, the ecological fallacy in the interpretation of results due to the heterogeneity in the characteristics of the studies (methodological diversity) and also to the population samples used in each of them (clinical diversity) [82]. And, third, the concepts of bioactivation and biofunctionalization of surfaces are not always clear in the different studies included in this systematic review, leading to great heterogeneity of results.

This heterogeneity of results could be due to the surface coating process and the different methods used to evaluate the bone–implant surface contact: mechanical, histomorphometric, and radiological (ISQ, RTT, RE, SF, BIC, and BA). Although Yang and colleagues [83] found greater osseointegration and bone apposition using an electrochemical process in the modification of surfaces, the limited information provided by most of the studies makes it difficult to determine the best methods of surface modification. Finally, another important aspect regarding the limitations of our study was the publication bias, and therefore we are aware that our conclusions can only be applied to the sample of included studies.

Therefore, to determine the effect of bioactive and biofunctionalized surfaces on implant osseointegration, it is necessary to reduce the risk for bias of the studies, eliminate confounding factors, and establish a clear definition of adequate parameters, all of this aimed at obtaining the results that might be useful in a wide range of clinical applications so that scientific evidence may support the practice of clinical dentistry.

The purpose of this review was to assess the impact of bioactive surface modification on implant osseointegration. However, it proved difficult to conclude that such modifications might be beneficial in terms of osseointegration, mainly because the risk of bias was high in most of the studies included and their analysis was complicated and problematic, hampering the interpretation of results.

## 5. Conclusions

The effect of bioactive modifications of dental implant surfaces is not always beneficial for osseointegration, although certain biomolecules used for coating seem to influence early peri-implant bone formation. All the materials proposed in the different studies included in this systematic review to modify the implant surfaces of Ti and improve its osseointegration have different advantages and limitations in terms of mechanical properties, biocompatibility, and osseointegration potential. Therefore, long-term clinical trials are required to validate the success of implants using this type of biomolecular coating. On the other hand, it should be noted that the results obtained using animal models cannot always be extrapolated to human clinical reality.

## Figures and Tables

**Table 1:** Checklist of the STROBE (STrengthening the Reporting of OBservational studies in Epidemiology) criteria reported in the human studies.

Studies	Gursoytrak, B., Ataoglu, H. 2020 [23]	Malchiodi et al. 2011 [24]	Mistry et al. 2010 [25]
Section and item			
1. Title and abstract	1	1	1
Introduction			
2.Background	1	1	1
3. Objectives	1	1	1
Methods			
4. Study design	1	1	1
5. Setting	1	1	1
6. Participants	1	1	1
7. Variables	1	0	1
8. Data sources/measurement	1	1	1
9. Bias	0	0	0
10. Study size	1	1	1
11.Quantitative variables	1	1	1
12. Statistical methods	1	0	1
Results			
13. Participants	1	1	1
14. Descriptive data	0	0	0
15. Outcome data	1	1	1
16. Main results	1	1	1
17. Other analyses	0	0	1
Discussion			
18. Key results	1	1	1
19. Limitations	0	0	0
20. Interpretation	0	0	0
21. Generalizability	0	0	0
Other information			
22. Funding	1	0	1
Total score	16	13	17

Mode Value: 15.13 ± 2.08. Each item was attributed a score of “0” (not reported) or “1” (reported). The total score of each of the included studies was also recorded.

**Table 2:** Checklist of the ARRIVE (Animal Research: Reporting of In Vivo Experiments) criteria reported in the included studies.

Studies	Cho et al. 2019 [26]	Łukas Zewska-Kuska et al. 2019 [27]	Romero-Ruiz et al. 2019 [28]	Lee et al. 2019 [29]	Thiem et al. 2019 [30]	Chan et al. 2018 [31]	Romero-Gavilan et al. 2018 [32]	Huanhuan et al. 2017 [33]	Herrero-Climent et al.2018 [34]	Su et al. 2017 [35]	van Oirschot et al. 2014 [36]	Galli et al. 2014 [37]	Kang et al. 2013 [38]	Gobbato et al. 2012 [39]	Choi et al. 2012 [40]	Aparicio et al. 2011 [41]	Diefenbeck et al. 2011 [42]	Azen ha et al. 2010 [43]	Lutz et al. 2010 [44]	Quaranta et al. 2010 [45]	Barros et al. 2009 [46]	Granato et al. 2011 [47]	Fawzy et al. 2008 [48]	Faeda et al. 2009 [49]	Alexander et al. 2009 [50]	Germanier et al. 2006 [51]	Teixeira et al. 2012 [52]
1. Title	1	1	1	1	1	1	1	1	1	1	1	1	1	1	1	1	1	1	1	1	1	1	1	1	1	1	1
Abstract																											
2. Species	1	1	1	1	1	1	1	1	1	1	1	1	1	1	1	1	1	1	1	1	1	1	1	1	1	1	1
3. Key finding	1	1	1	1	1	1	1	1	1	1	1	1	1	1	1	1	1	1	1	1	1	1	1	1	1	1	1
Introduction																											
4. Background	1	1	1	1	1	1	1	1	1	1	1	1	1	1	1	1	1	1	1	1	1	1	1	1	1	1	1
5. Reasons for animal models	1	1	1	0	1	1	1	1	0	0	0	0	0	0	0	0	0	0	0	0	0	0	0	0	0	0	0
6. Objectives	1	1	1	1	1	1	1	1	1	1	1	1	1	1	1	1	1	1	1	1	1	1	1	1	1	1	1
Methods																											
7. Ethical statement	1	1	1	1	1	1	1	1	1	1	1	1	1	1	1	1	1	1	1	1	0	1	1	1	1	1	1
8. Study design	1	1	1	1	1	1	1	1	1	1	1	1	1	1	1	1	1	1	1	1	1	1	1	1	1	1	1
9. Experimental procedures	1	1	1	1	1	1	1	1	1	1	1	1	1	1	1	1	1	1	1	1	1	1	1	1	1	1	1
10. Experimental animals	1	1	1	1	1	1	1	1	1	1	1	1	1	1	1	1	1	1	1	1	1	1	1	1	1	1	1
11. Accommodation and handling of animals	1	1	1	0	1	1	0	0	0	0	0	0	0	0	0	1	0	0	0	0	0	0	0	0	1	0	0
12. Sample size	1	1	1	1	1	1	1	1	1	1	1	1	1	1	1	1	1	1	1	1	1	1	1	1	1	1	1
13. Assignment of animals to experimental groups	1	1	1	0	0	0	0	0	0	0	0	0	0	0	0	1	0	0	1	1	0	0	1	1	1	1	1
14. Anesthesia	1	1	1	1	1	1	1	1	1	1	1	1	1	1	1	1	1	1	1	1	1	1	1	1	1	1	1
15. Statistical methods	1	1	1	1	1	1	1	1	1	1	1	1	1	1	1	1	1	1	1	1	1	1	1	1	1	1	1
Results																											
16. Experimental results	1	1	1	1	1	1	1	1	1	1	1	1	1	1	1	1	1	1	1	1	1	1	1	1	1	1	1
17. Results and estimation	1	1	1	1	1	1	1	1	1	1	1	1	1	1	1	1	1	1	1	1	1	1	1	1	1	1	0
Discussion																											
18. Interpretation and scientific implications	1	1	1	1	1	1	1	1	1	1	1	1	1	1	1	1	1	1	1	1	1	1	1	1	1	1	1
19. 3Rs reported	0	0	0	0	0	0	0	0	0	0	0	0	0	0	0	0	0	0	0	0	0	0	0	0	0	0	0
20. Adverse events	0	0	0	0	0	0	1	0	0	0	0	0	0	0	0	0	0	0	0	1	0	0	0	0	0	0	0
21. Study limitations	0	0	0	0	1	0	0	1	0	0	0	0	0	0	1	0	0	0	0	0	0	0	0	0	0	0	0
22.Generalization/applicability	1	1	1	1	1	1	1	1	0	1	0	1	0	1	1	1	1	1	1	1	1	1	1	1	1	1	1
23. Funding	1	1	1	1	1	1	1	1	1	0	1	0	1	0	1	1	1	1	1	1	1	1	0	0	1	1	1
Total Score	20	20	20	17	20	19	18	19	16	16	16	16	16	16	18	19	17	17	18	19	16	17	17	17	19	18	17

Mode Value: 17.7 ± 1.4. Each item was attributed a score of “0” (not reported) or “1” (reported). The total score of each of the included studies was also recorded.

**Table 3:** Animal studies.

Studies	Animals	Surface Preparation	Number of Implants	Implantation Sites	Tracing (Weeks)	Conclusions	BIC Values		BA Values		ISQ Values		RTT Values		RE Values		SF Values	
							Test	Control	Test	Control	Test	Control	Test	Control	Test	Control	Test	Control
**Cho et al. 2019** [26]	New Zealand white rabbit model	A human vitronectin-derived peptide, VnP-16	16	Tibia	2 weeks	VnP-16 reinforces the osteogenic potential of an SLA titanium dental implant when this peptide is applied to the SLA surface.	NR	NR	**61.5 ± 10.6%** ****		NR	NR	NR	NR	NR	**NR**	NR	NR
**Łukaszewska-Kuska et al. 2019** [27]	New Zealand white rabbit model	Hydroxyapatite (HA) coating	20	Tibia	2 weeks	The HA coating reported herein was found to have chemical and physical properties which appear to improve osseointegration compared to grit-blasted implants.	NR	NR	NR	69.85 **±** 2.05% ****	NR	NR	NR	NR	NR	NR	NR	NR
**Romero-Ruiz et al. 2019** [28]	Minipig model	ContacTi^®^ (alumina particle bombardment of titanium bioactivated when treated thermochemically)	12	Jaw, premolar and molar area.	8 weeks	The surface ContacTi^®^ showed remarkable results in terms of the osseointegration process.	NR	NR	NR	73.5 **±** 1.3% ****	NR	NR	NR	NR	NR	NR	NR	NR
**Lee et al. 2019** [29]	Dog model	(IS-III Bioactive^®^) SLA with HA nanocoating	9	Jaw, the second, the third, and the fourth premolars area	4 weeks	Osteoblasts might become more activated with the use of an HA-coated surface.	77.28 ± 11.22%	68.80 ± 10.67%	44.94 ± 17.69%	36.53 ± 13.72%	NR	NR	NR	NR			NR	NR
**Thiem et al. 2019** [30]	New Zealand white rabbit model	Nanocrystalline SiO_2_–HA coating	36	Femur	2 and 4 weeks	Distance osteogenesis does not seem to become affected by a bioactive SiO_2_–HA surface coating.	2 weeks, 66 ± 3% ** 4 weeks **, 65 ± 2%	2 weeks, 42 ± 1% 4 weeks, 44 ± 1%	NR	NR	NR	NR	NR	NR	NR	NR	NR	NR
**Chan et al. 2018** [31]	New Zealand white rabbit model	Bioactive glass fiber-reinforced composite (GFRC)	12	Femur	8 weeks	Histological evaluation revealed more newly formed bone regeneration in the GFRC implant group during the initial healing period.	37.9 ± 1.6% **	37.1 ± 5.9%	NR	NR	NR	NR	NR	NR	NR	NR	NR	NR
**Romero-Gavilan et al. 2018** [32]	New Zealand white rabbit model	Silica hybrid sol-gel coating applied onto the Ti substrate (35M35G30T)	10	Tibia	4 weeks	Implants coated with the 35M35G30T coating demonstrated a clear increase in inflammatory activity, surely due to an associated, natural, and controlled immune response.	NR	NR	NR	NR	NR	NR	NR	NR	40.4 ± 27.9% ***	44.4 ± 21.6%	NR	NR
**Huanhuan et al. 2017** [33]	Rat model	Sr overcoated acid-etched titanium implant (SLA)	20	Tibia	2 and 8 weeks	The Sr–SLA surface showed increased BIC (Bone of Implant Contact) and new bone apposition around the implants. The result indicated that the Sr–SLA surface has an effect that improves early osseointegration.	2 weeks **, 28.76 ± 8.44% 8 weeks **, 62.5 ± 35.78%	2 weeks, 22.57 ± 6.29% 8 weeks, 45.54 ± 9.59%	2 weeks, 12.02 ± 4.45% 8 weeks, 41.62 ± 7.75%	2 weeks, 9.82 ± 3.49 8 weeks, 29.55 ± 5.53%	NR	NR	NR	NR	NR	NR	NR	NR
**Herrero-Climent et al. 2018** [34]	Minipig model	Blasting of combined abrasive Al_2_O_3_ particles with thermochemical treatment (ContacTi^®^)	20	Maxillae	2, 4, and 8 weeks	The ContacTi^®^ surface achieved faster growth of hard tissues around the implants compared to the blasting surface, and for all the histomorphometric parameters evaluated, the values were higher.	2 weeks, 49.02 ± 26.3% 4 weeks **, 83.20 ± 8.12% 8 weeks **, 85.58 ± 3.81%	2 weeks, 39.32 ± 2.48% 4 weeks, 46.53 ± 9.81% 8 weeks, 46.20 ± 3.54%	NR	NR	NR	NR	NR	NR	NR	NR	NR	NR
**Su et al. 2017** [35]	Rat model	Ca nanosurface	24	Femur	8 weeks	Nanostructure modification with incorporation of Ca^2+^ ions has a synergistic effect on the bone response to the implant.	NR	NR	NR	NR	NR	NR	NR	NR	20.58 ± 3.02% **	70.25 ± 4.53%	NR	NR
**van Oirschot et al. 2014** [36]	Goat model	HA coating	20	Iliac crest	4 weeks	HA coating enhanced the biological properties compared to grit-blasted/acid-etched implants.	57.5 ± 8.5% **	40.7 ± 13.2%	43.6 ± 9.0% ****	32.0 ± 10.4%	NR	NR	NR	NR	NR	NR	NR	NR
**Galli et al. 2014** [37]	New Zealand white rabbit model	Coated with thin films of mesoporous TiO_2_ having pore diameters of 6 nm and loaded with magnesium	20	Tibia	3 weeks	Local release of magnesium from implant surfaces improves implant retention in the early healing stage (3 weeks after implantation).	15.2 ± 17.6% ****	8.51 ± 3.4%	66.61 ± 10.3% ****	74.4 ± 15.2%	NR	NR	NR	NR	NR	NR	NR	NR
**Kang et al. 2013** [38]	New Zealand white rabbit model	Laminin-2-derived peptide	12	Tibia	Not reported	Titanium implants coated with a laminin-2-derived peptide can promote osseointegration by accelerating new bone formation in vivo.	‡‡	NR	NR	NR	NR	NR	NR	NR	NR	NR	NR	NR
**Gobbato et al. 2012** [39]	New Zealand white rabbit model	Ca–Ph-coated (BAE-2)	16	Tibia	1, 3, and 13 weeks	The bioactive BAE-2 implant surface provided healthy bone remodeling at 21 days of healing.	‡‡	NR	NR	NR	NR	NR	NR	NR	NR	NR	NR	NR
**Choi et al. 2012** [40]	New Zealand white rabbit model	Bioactive fluoride-modified	10	Tibia	2 weeks	The surface modified with bioactive fluoride does not show superiority in the early bone response.	42.6 ± 4.0% ****	36.0 ± 5.4%	47.0 ± 5.4% ****	47.4 ± 3.4%						NR		
**Aparicio et el. 2011** [41]	Minipig model	Micro-rough acid-etched (2Step)	32	Mandible and maxilla	2, 4, 6, and 10 weeks	The 2Step treatment produced micro-rough and bioactive implants that accelerated bone tissue regeneration and increased mechanical retention in the bone bed at short periods of implantation.	‡	NR	NR	NR	NR	NR	NR	NR	NR	NR	NR	NR
**Diefenbeck et al. 2011** [42]	Rat model	Plasma chemical oxidation (Ca–Ph) (TiOB surface)	128	Tibia	3 and 8 weeks	The bioactive TiOB surface has a positive effect on implant anchorage by enhancing the bone–implant contact.	NR	NR	3 weeks, 47.4 ± 11.5% ** 8 weeks, 60.8 ± 7.8% **	3 weeks, 27.5 ± 4.40% 8 weeks, 69.0 ± 6.04%	NR	NR	NR	NR	NR	NR	NR	NR
**Azenha et al. 2010** [43]	New Zealand white rabbit model	SiO_2_–P_2_O_5_–Na_2_O, CaO, and Bioglass^®^45S5	64	Femur	8 and 12 weeks	All tested materials are biocompatible and are suitable to be used in clinical dentistry.	8 weeks, 93 ± 6.55% **** 12 weeks, 90 ± 9% ****	8 weeks, 87 ± 8% 12 weeks, 92 ± 8%	NR	NR	NR	NR	NR	NR	NR	NR	NR	NR
**Lutz et al. 2010** [44]	Pig model	The experimental implants were coated with HA and additionally with an active biomimetic peptide (P-15)	12	Jaw, premolar and molar area.	2 and 4 weeks	Biofunctionalization of the implant surface with a biomimetic active peptide leads to significantly increased BIC rates at 14 and 30 days and higher peri-implant bone density at 30 days.	2 weeks, 76.7 ± 26.1% 4 weeks, 75.8 ± 23.9%	2 weeks, 63.8 ± 28.1% 4 weeks, 75.8 ± 23.9%	NR	NR	NR	NR	NR	NR	NR	NR	NR	NR
**Quaranta et al. 2010** [45]	New Zealand white rabbit model	Plasma-sprayed calcium-phosphate (PSCa–Ph)	48	Femur	2, 4, and 8 weeks	Bioactive ceramic coatings were biocompatible and osteoconductive. However, the early bone response was favored by the presence of the thicker PSCaP coating.	3 weeks, 27.1 ± 1.1% 4 weeks, 43.0 ± 3.0% 8 weeks, 61.0 ± 4.5% **	3 weeks, 23.0 ± 0.2% 4 weeks, 31.5 ± 2.4% 8 weeks, 46.0 ± 4.1%	NR	NR	NR	NR	NR	NR	NR	NR	NR	NR
**Barros et al. 2009** [46]	Dog model	Application of a thin HA + bioactive peptide coating	32	Mandibular premolar area	8 weeks	Biofunctionalization of the implant surface interferes with bone apposition around titanium implants, especially in terms of bone density.	47.0 ± 16.8% ****	41.4 ± 18.7%	NR	NR	NR	NR	NR	NR	NR	NR	NR	NR
**Granato et al. 2011** [47]	Dog model	Bioactive ceramic coating deposition on an alumina-blasted/acid-etched surface	16	Tibia	2 and 4 weeks	A thin bioactive ceramic coating on the implant surface did not affect BIC, but positively affected the biomechanical fixation of the implant.	2 weeks, 71.70 ± 20.37% 4 weeks, 75.70 ± 18.20% ****	2 weeks, 79.02 ± 16.02% 4 weeks, 86.99 ± 8.40%	NR	NR	NR	NR	NR	NR	NR	NR	NR	NR
**Fawzy et al. 2008** [48]	New Zealand white rabbit model	NaOH/heat treatment	46	Tibia	2, 4, and 8 weeks	The sodium removal treatment was shown to be effective in improving the early resistance of the bone–implant interface.	NR	NR	NR	NR	NR	NR	NR	NR	NR	NR	2 weeks, 91.12 ± 36.57% 4 weeks, 240.72 ± 97.41% 8 weeks, 562.45 ± 132.93% **	2 weeks, 61.50 ± 28.15% 4 weeks, 214.56 ± 61.31% 8 weeks, 508.20 ± 111.78%
**Faeda et al. 2009** [49]	New Zealand white rabbit model	HA coatings	96	Tibia	4, 8, and 12 weeks	Implants with the HA biomimetic coating can shorten the healing period of implants by increasing the implant–bone interaction during the first 2 months after implant placement.	NR	NR	NR	NR	NR	NR	NR	NR	2 weeks, 55.42 ± 12.86% 8 weeks, 24.0 ± 6.34% 12 weeks, 33.85 ± 6.28% **	2 weeks, 23.28 ± 4.46% 8 weeks, 63.71 ± 14.79% 12 weeks, 64.0 ± 18.05%	NR	NR
**Alexander et al. 2009** [50]	Non-human primate model	Ca–Ph surface	25	Lower jaw	30 weeks	Implant coating with ultra-fine calcium phosphate favors osteoconductive properties in the early phase with the avoidance of adverse reactions against the material during the later stages of osseointegration.	74.9 ± 9.8% ****	73.2 ± 17%	NR	NR	NR	NR	NR	NR	NR	NR	NR	NR
**Germanier et al. 2006** [51]	Miniature pig model	Arg–Gly–Asp (RGD) peptide-modified polymer (PLL-g-PEG/PEG–RGD) (poly(L lysine)- graft-poly(ethylene glycol/ poly(ethylene glycol- Arg–Gly–Asp) PLL (120 L-lysine units) PEG (47 ethylene glycol units)	48	Anterior maxilla	2 and 4 weeks	Significant enhancement of new bone apposition to the RGD-functionalized SLA surface during the very early stages.	2 weeks, 61.68 ± 4.21% *** 4 weeks, 62.52 ± 8.04% ***	2 weeks, 43.62 ± 10.79% 4 weeks, 62.46 ± 6.37%	NR	NR	NR	NR	NR	NR	NR	NR	NR	NR
**Teixeira et al. 2012** [52]	Dog model	Alumina-blasted and acid-etched (AB/AE) surface	36	Center of the radius diaphysis	2 and 4 weeks	Dental implant treatment with textured surfaces with argon plasma produced substantial improvements in biomechanical fixation in the early stages of implantation.	2 weeks ‡ 4 weeks ‡	2 weeks ‡ 4 weeks ‡	NR	NR	NR	NR	NR	NR	NR	NR	NR	NR

HA (hydroxyapatite); SIO_2_ (silicon dioxide); Sr (strontium); SLA (sandblasted with long-grit corundum followed by acid etching with sulfuric and hydrochloric acid); Al_2_O_3_ (aluminum oxide); Ca (calcium); Ta (tantalum); P (phosphorus); P_2_O_5_ (Phosphorus Oxide); CaO (Calcium Oxide); NaOH (Sodium Hydroxide). BIC (bone implant contact); BA (bone area); ISQ (implant stability quotient); RTT (removal torque test); RE (radiological evaluation); SF (shear force); NR, not reported; ‡ Reported in a figure; ‡‡ Reported in a histological image. Significance: * *p* < 0.01, ** *p* < 0.05, *** *p* < 0.001, **** *p* > 0.01.

**Table 4:** Human studies.

Studies	Type of Study	Surface Preparation	Number of Implants	Implantation Sites	Tracing (Weeks)	Conclusions	BIC Values		ISQ Values	
							Test	Control	Test	Control
Gursoytrak, B., Ataoglu, H. 2020 [23]	Randomized clinical study	Alkali-modified (bioactive) and sandblasted surfaces	50 (2 groups)	Mandibular molar area	2, 6, and 12 weeks	No significant differences. The ISQ of the bioactive implants that exhibit high primary stability fell more than those of the implants with sandblasted surfaces at 2 and 6 weeks after the operation; both types of implants produced similar clinical results at 12 weeks post-operation.	NR	NR	2 weeks, 73.68 ± 3.84% 6 weeks, 69.8 ± 4.61% 12 weeks, 73.40 ± 4.30% *	2 weeks, 72.91 ± 4.63%6 weeks, 71.36 ± 7.42 12 weeks, 72.15 ± 3.39%
Malchiodi et al. 2011 [24]	Case series	Resorbable calcium phosphate (CaP) coating made of brushite (FBR)	3	Posterior mandible	8, 10, and 12 weeks	Immediately loaded FBR implants placed in the posterior jaw can achieve osseointegration within 6–12 weeks of loading.	54.4 ± 3.74% ****	70.1 ± 2.16%	NR	NR
Mistry et al. 2010 [25]	Not reported	Bioactive glass (BG) coating	62	Anterior maxilla and anterior mandible	12 months	Overall results showed that BG-coated implants are as successful as HA-coated implants in achieving osseointegration.	Bioactive glass group: 6 months, 0.93 ± 0.26% 12 months, 0.78 ± 0.42%	HA group: 6 months, 0.92 ± 0.30% 12 months, 0.82 ± 0.40% **	NR	NR

BIC (bone implant contact); ISQ (implant stability quotient); NR, not reported. Significance: * *p* < 0.01, ** *p* < 0.05, **** *p* > 0.01.

## Supplementary Materials

The following are available online at https://www.mdpi.com/2077-0383/9/7/2047/s1, Table S1: PRISMA Checklist.

## Abbreviations

BIC	bone implant contact
BA	bone area
RTT	removal torque test
RE	radiological evaluation
SF	shear force
ISQ	implant stability quotient
HA	hydroxyapatite
SiO_2_	silicon dioxide
Sr	strontium
SLA	sandblasted with long-grit corundum followed by acid etching with sulfuric and hydrochloric acid
Al_2_O_3_	aluminum oxide
Ca	calcium
Ta	tantalum
Ti	titanium
P	phosphorus
Ar	argon
P_2_O_5_	phosphorus oxide
CaO	calcium oxide
NaOH	sodium hydroxide
CaP	calcium phosphate
