# Supplementary material for: Bioactive Surfaces vs. Conventional Surfaces in Titanium Dental Implants: A Comparative Systematic Review
Source: J Clin Med. 2020 Jun 29;9(7):2047. doi: 10.3390/jcm9072047 (PMC7408888; doi:10.3390/jcm9072047)
Supplement: Supplementary file 1 [file jcm-09-02047-s001.pdf]

Table S1. PRISMA Checklist.

Section/topic	#	Checklist item	Reported on page #
**TITLE**			
Title	1	Identify the report as a systematic review, meta-analysis, or both.	1
**ABSTRACT**			
Structured summary	2	Provide a structured summary including, as applicable, background; objectives; data sources; study eligibility criteria, participants, and interventions; study appraisal and synthesis methods; results; limitations; conclusions and implications of key findings; systematic review registration number.	1
**INTRODUCTION**			
Rationale	3	Describe the rationale for the review in the context of what is already known.	2
**METHODS**			
Objectives	4	Provide an explicit statement of questions being addressed with reference to participants, interventions, comparisons, outcomes, and study design (PICOS).	4
Protocol and registration	5	Indicate if a review protocol exists, if and where it can be accessed (e.g., a web address), and, if available, provide registration information including registration number.	4
Eligibility criteria	6	Specify study characteristics (e.g., PICOS, length of follow-up) and report characteristics (e.g., years considered, language, publication status) used as criteria for eligibility, giving rationale.	4
Information sources	7	Describe all information sources (e.g., databases with dates of coverage, contact with study authors to identify additional studies) in the search and the date last searched.	4
Search	8	Present a full electronic search strategy for at least one database, including any limits used, so that it could be repeated.	5

Study selection	9	State the process for selecting studies (i.e., screening, eligibility, included in a systematic review, and, if applicable, included in a meta-analysis).	4
Data collection process	10	Describe the method of data extraction from reports (e.g., piloted forms, independently, in duplicate) and any processes for obtaining and confirming data from investigators.	5
Data items	11	List and define all the variables for which data were sought (e.g., PICOS, funding sources) and any assumptions and simplifications made.	4
Risk of bias in individual studies	12	Describe the methods used for assessing risk of bias of individual studies (including specification of whether this was done at the study or outcome level) and how this information is to be used in any data synthesis.	5
Summary measures	13	State the principal summary measures (e.g., risk ratio, difference in means).	5
Synthesis of results	14	Describe the methods of handling data and combining results of studies, if done, including measures of consistency (e.g., I^2) for each meta-analysis.	18

From: Moher, D., Liberati, A., Tetzlaff, J., Altman, DG., the PRISMA Group (2009). Preferred Reporting Items for Systematic Reviews and Meta-Analyses: the PRISMA Statement. PLoS Med 6(7): e1000097. doi:10.1371/journal.pmed100009
